# Exploiting the Bragg Mirror Effect of TiO_2_ Nanotube Photonic Crystals for Promoting Photoelectrochemical Water Splitting

**DOI:** 10.3390/nano14211695

**Published:** 2024-10-23

**Authors:** Ming Meng, Hucheng Zhou, Jing Yang, Liwei Wang, Honglei Yuan, Yanling Hao, Zhixing Gan

**Affiliations:** 1 School of Physics and Telecommunication Engineering, Zhoukou Normal University, Zhoukou 466001, China; mengming@zknu.edu.cn (M.M.); 20182013@zknu.edu.cn (H.Z.); yangjing0410@zju.edu.cn (J.Y.); wangliwei@zknu.edu.cn (L.W.); yhl@zknu.edu.cn (H.Y.); 2 Key Laboratory for Micro-Nano Functional Materials of Qianxinan, Minzu Normal University of Xingyi, Xingyi 562400, China; 3 Center for Future Optoelectronic Functional Materials, Nanjing Normal University, Nanjing 210023, China; 4 School of Computer and Electronic Information/School of Artificial Intelligence, Nanjing Normal University, Nanjing 210023, China

**Keywords:** TiO_2_ nanotube, photonic crystals, Bragg mirror effect, photoelectrochemical water splitting

## Abstract

Exploiting the Bragg mirror effect of photonic crystal photoelectrode is desperately desired for photoelectrochemical water splitting. Herein, a novel TiO_2_ nanotube photonic crystal bi-layer structure consisting of a top nanotube layer and a bottom nanotube photonic crystal layer is presented. In this architecture, the photonic bandgap of bottom TiO_2_ nanotube photonic crystals can be precisely adjusted by modulating the anodization parameters. When the photonic bandgap of bottom TiO_2_ nanotube photonic crystals overlaps with the electronic bandgap of TiO_2_, the bottom TiO_2_ nanotube photonic crystal layer will act as a Bragg mirror, leading to the boosted ultraviolet light absorption of the top TiO_2_ nanotube layer. Benefiting from the promoted UV light absorption, the TiO_2_ NT-115-NTPC yields a photocurrent density of 1.4 mA/cm^2^ at 0.22 V vs. Ag/AgCl with a Faradic efficiency of 100%, nearly two times higher than that of conventional TiO_2_ nanotube arrays. Furthermore, incident photon-to-current conversion efficiency is also promoted within ultraviolet light region. This research offers an effective strategy for improving the performance of photoelectrochemical water splitting through intensifying the light–matter interaction.

## 1. Introduction

Photoelectrochemical (PEC) water splitting has been regarded as a desirable avenue to exploit the abundant and sustainable solar energy by directly transforming the incident light into hydrogen fuel [1,2,3,4,5,6,7]. Among various PEC materials that can work as photoelectrodes, TiO_2_ nanotube array (TiO_2_ NTA), vertically grown on a conductive Ti foil by electrochemical anodization, distinguishes itself owing to its multifold unique merits including large internal and external surface areas, unidirectional electrical channel, and outstanding adhesion with Ti foil [8,9,10]. Unfortunately, the pristine TiO_2_ NTA has a wide bandgap of 3.2 eV, which means that it still suffers from limited photoconversion efficiency even in the ultraviolet (UV) region [11,12,13]. Until now, element doping and narrow bandgap semiconductor coupling have been the two major strategies for extending the visible light absorption of TiO_2_ [14,15,16,17]. However, these strategies bear many adverse effects, such as limited visible light response, increased carrier recombination centers, decreased incident photon-to-electron conversion efficiency (IPCE) in the UV region and the redox ability of the photogenerated charges [18,19,20,21]. Accordingly, gaining the utmost out of the UV light may be another promising approach to promote the PEC performance of TiO_2_ NTA.

Recently, introducing a photonic crystal nanostructure into photocatalysts furnishes a new emerging route of strengthening light–matter interaction to improve light absorption [22,23,24]. The photonic crystal photocatalysts possess a periodic dielectric structure, which endows them with a photonic bandgap (PBG) for a certain frequency of photons [25,26,27]. To be specific, the group velocity of the photons with the frequency near the PBG edges can be significantly slowed, referred as the slow photon effect [28,29,30,31]. In addition, the photons with the frequency range of PBG are totally reflected and cannot propagate in the photonic crystal structure due to Bragg reflection (called the Bragg mirror effect) [32,33,34]. Obviously, the slow photon effect and Bragg mirror effect hold immense promise for intensifying light–material interaction, resulting in an amplified light absorption and photoelectrochemical reaction. Yet, to date, the existing investigations have mainly focused on the utilization of the slow photon effect in a single layer of three-dimensional (3D) TiO_2_ inverse opal structures [13,28,35,36,37], which cannot use the reflected light at the PBG of TiO_2_ to promote PEC performance in the UV region. 

Apart from the 3D TiO_2_ inverse opal structures, novel TiO_2_ nanotube photonic crystals (TiO_2_ NTPCs) with periodicities along the axial direction of nanotube have been successfully fabricated by a simple periodic current pulse anodization process [32,38,39]. Furthermore, the PBGs of TiO_2_ NTPCs can be continuously adjusted through controlling the fabrication parameters [32,38,39]. Undoubtedly, after constructing the TiO_2_ NTPC bi-layer structure consisting of a top nanotube (NT), which functions as an absorbing layer, and the bottom NTPC with PBG overlapping with an electronic bandgap of TiO_2_ that acts as Bragg mirror layer, the interaction of top TiO_2_ NT layer with reflected UV light should be greatly boosted, which could enhance its PEC performance in the UV region. Nevertheless, there is as yet no investigation available on the TiO_2_ NTPC bi-layer structure focusing on the correlation between the Bragg mirror effect and PEC performance. This also implies that the underlying physical mechanism also remains unclear. 

Herein, the novel TiO_2_ NTPC bi-layer structure consisting of a top NT layer and a bottom NTPC layer was designed and fabricated for PEC water splitting. As expected, the TiO_2_ NTPC bi-layer structure, with the PBG of bottom NTPC overlapping with an electronic bandgap of TiO_2_, yielded a photocurrent density of 1.4 mA/cm^2^ at 0.22 V vs. Ag/AgCl with Faradic efficiency of 100%, nearly two times higher than that of conventional TiO_2_ NTA. Furthermore, IPCE was also promoted within the UV light region. Such remarkable enhancement of PEC water splitting activity was primarily derived from the fact that the bottom NTPC layer can function as a Bragg mirror that can promote the interaction of top TiO_2_ NT layer with the reflected UV light, thus leading to the boosted UV light absorption of the top TiO_2_ NT layer. This work offers an effective strategy for improving the performance of PEC water splitting through intensifying light–matter interaction.

## 2. Materials and Methods

### 2.1. Materials

Ammonium fluoride (NH_4_F), Ethylene glycol and Sodium hydroxide (NaOH) were obtained from Sinopharm Chemical Reagent Co., Ltd., Shanghai, China. Ti foils (0.25 mm thick, 99.8% purity) were purchased from Anping Anheng Wire Mesh Co., Ltd., Hengshui, China. All the chemicals were utilized as received without any further purification.

### 2.2. Fabrication of the TiO_2_ NTPC Bi-Layer Structure

The TiO_2_ NTPC bi-layer structure consisting of a top nanotube (NT) layer and a bottom NTPC layer was prepared by successive two-step anodization. Specifically, the Ti foils with sizes of 1.5 cm × 1 cm were firstly pre-treated by anodization at 60 V for 1 h, using glycol aqueous solution containing 0.5 wt% NH_4_F and 2 vol% DI H_2_O. Then, the as-grown TiO_2_ NT was ultrasonically removed in deionized (DI) H_2_O. After that, the pre-treated Ti foils were subjected to a successive two-step anodization process composed of a constant current anodization part and subsequently a periodic current pulse anodization part. During the first step, the constant current was maintained at 7 mA/cm^2^ for 10 min to form the top TiO_2_ NT layer. During the second step, the periodic current pulse anodization with high current (HC, $J_{HC}=7{\;\mathrm{mA}/\mathrm{cm}}^{2}$) and low current (LC, $J_{HC}=0{\;\mathrm{mA}/\mathrm{cm}}^{2}$) was employed to fabricate the bottom TiO_2_ NTPC layer. The time duration of the HC pulse was controlled from 120 to 180 s, while the time duration of the LC pulse was fixed at 180 s to tailor the lattice constant of NTPC. Finally, the bi-layer structures were annealed at 450 °C in air for 2 h to obtain anatase TiO_2_. In addition, the single layer TiO_2_ NTPC with the same thickness as the bottom NTPC layer of the bi-layer was also prepared by constant current anodization.

### 2.3. Characterization

The morphologies, microstructures and crystal structures of the as-prepared samples were inspected by field-emission scanning electron microscopy (FE-SEM, S4800, Hitachi Ltd., Tokyo, Japan), field-emission transmission electron microscopy (FE-TEM, JEM-2100, JEOL Ltd., Tokyo, Japan), and X-ray powder diffractometry (XRD, Xpert, Philips, Amsterdam, The Netherlands). The diffuse reflectance spectra were recorded by a VARIAN Cary5000 spectrophotometer (Varian, CA, USA). The X-ray photoelectron spectroscopy (XPS) data were collected by a PHI 5000 Versaprobe (Ulvac-Phi, Kanagawa, Japan).

### 2.4. Photoelectrochemical Measurements

Photoelectrochemical measurements were performed in a three-electrode system connected to a CHI 660E electrochemical workstation (CH Instrument, Chenhua Ltd., Shanghai, China) utilizing the as-prepared samples with an exposed area of 1 cm^2^ as the working electrode, the Pt mesh as the counter electrode, and the Ag/AgCl (3 mol/L KCl-filled) as the reference electrode. The 1 M NaOH (pH = 13.6) solution was electrolyte, which was purged with N_2_ (99.999%) flow for 1 h to remove dissolved oxygen. The illumination source was a 500 W Xe lamp (Solar 500, NBet Group Corp. Beijing, China) with a calibrated intensity of 100 mW/cm^2^, and a water filter was placed between the lamp and the electrochemical cell to eliminate the infrared heating of the electrolyte. The incident photon-to-current conversion efficiency (IPCE) measurements were conducted at an applied potential of 0.22 V vs. Ag/AgCl by means of a monochromatic system. During the PEC stability measurement, the photoelectrodes were biased at 0.22 V vs. Ag/AgCl. The amount of evolved oxygen was quantified by an Ocean Optics oxygen sensor system equipped with a FOXY probe (NeoFox Phase Measurement System), which was measured together with PEC stability.

## 3. Results and Discussion

### 3.1. Morphological Characterization of the TiO_2_ NTPC

**Figure 1:**
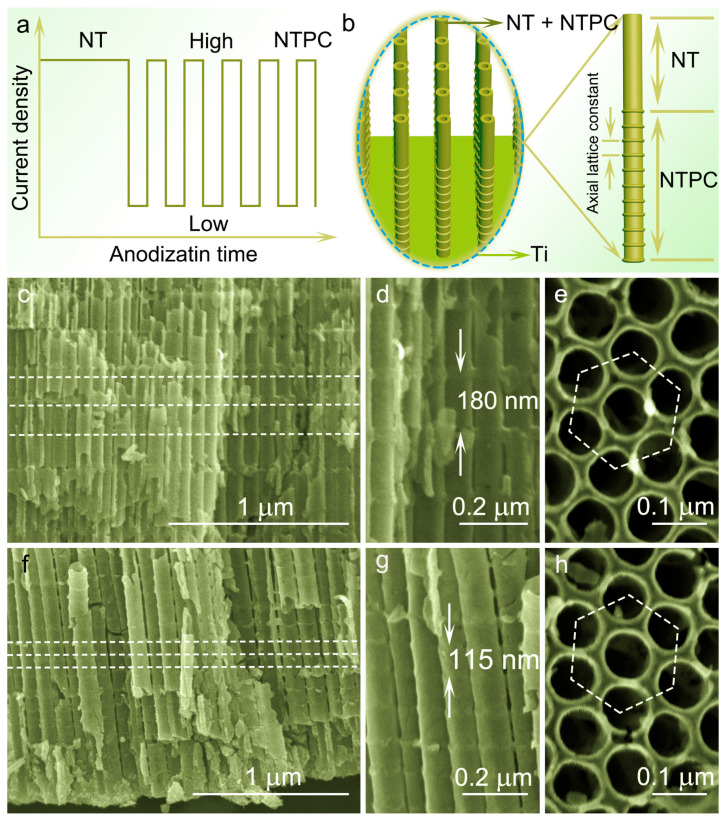
(**a**) A schematic illustration of the current–time curve of anodization and the TiO_2_ nanotube/nanotube photonic crystal bi-layer structure. (**b**) Schematic illustration of the TiO_2_ nanotube/nanotube photonic crystal bi-layer structure. (**c**–**e**) FE-SEM images of the TiO_2_ 180-NTPC. (**f**–**h**) FE-SEM images of the TiO_2_ 115-NTPC.

Figure 1a,b are a schematic illustration of the current–time curve of anodization and TiO_2_ nanotube photonic crystal (NTPC) bi-layer structure consisting of a top NT and a bottom NTPC. In brief, the single constant current anodization was first utilized to form the top smooth-walled TiO_2_ NT layer on the Ti foil. The as-grown sample was then subjected to periodic current pule anodization with high current and low current to form a TiO_2_ NTPC layer with periodicities along the axial direction beneath the TiO_2_ NT layer. The most crucial step in this work was to accurately modulate the lattice constant of the TiO_2_ NTPC for obtaining the desired featured. This could be realized by adjusting the duration of high current pulse anodization, since the lattice constant of the NTPC layer was almost linearly increased with it.

Figure 1c–e display the FE-SEM images of single-layer TiO_2_ NTPC fabricated by the periodic current pulse anodization with different durations of the HC pulse of 7 mA/cm^−2^. Obviously, a TiO_2_ NTPC presents a bamboo-shaped periodic structure in the axial direction of the NT. Such a periodic structure with alternating protrusive bamboo node layers and smooth-walled tube layers can result in a periodical refractive index change in the longitudinal direction, indicating that it can exhibit a structural modulated photonic bandgap (PBG). The length of the node and smooth-walled layer is the lattice constant of TiO_2_ NTPC, which increases from 115 nm to 180 nm for HC pulse durations of 120 s and 180 s, respectively. The corresponding samples are denoted as 115-NTPC and 180-NTPC, respectively. Additionally, these TiO_2_ NTPC have well-ordered and hexagonally arranged tubular structures with an average diameter of about 100 nm and a wall thickness of about 10 nm. More importantly, this allows the PBG of TiO_2_ NTPC to be adjusted at will to match the electronic bandgap of anatase TiO_2_.

### 3.2. Optical Obsorption Properties of the TiO_2_ NTPC Structure

**Figure 2:**
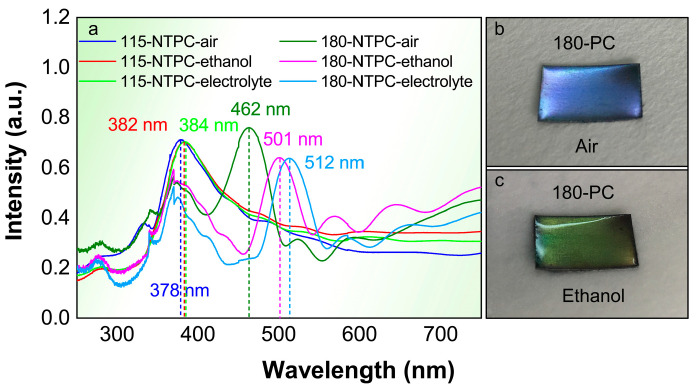
(**a**) The reflectance spectra of the TiO_2_ 180-NTPC and TiO_2_ 115-NTPC samples in air and infiltrated with ethanol and electrolytes, respectively. (**b**,**c**) Photographs of the TiO_2_ 180-NTPC samples in air and infiltrated with ethanol, respectively.

The reflectance spectra of TiO_2_ NTPCs with different lattice constants in air, ethanol and electrolytes are measured under normal incidence and are presented in Figure 2a. As the lattice constant increases from 115 nm to 180 nm, the refection peak of the TiO_2_ NTPC shifts to longer wavelengths. The positions of PBG of 115-NTPC with 15 periods and 180-NTPC with 15 periods are located at around 378 and 462 nm, respectively. The reflectance spectra of TiO_2_ NTPC are strongly influenced by the refractive index contrast [40,41]. When the TiO_2_ NTPC was put in ethanol, a remarkable red-shift of the reflection peaks could be observed, compared with that sample in air. The result is further reflected from the colors of the TiO_2_ NTPCs (Figure 2b,c and Figure S1). Specifically, after being infiltrated with ethanol, its color changes from purple to green. It should be noted that no color change in TiO_2_ 115-NTPC can be found, since its PBG is in the ultraviolet region (below 400 nm). When the refractive index contrast is further reduced by infiltration with liquid electrolyte (1 M NaOH), the reflection peaks of TiO_2_ NTPC shift to an even longer wavelength. Taking TiO_2_ 115-NTPC as an example, when the sample is immersed in electrolyte, the position of PBG shifts from 378 nm (air) to 384 (1 M NaOH) nm, which is very close to the electronic bandgap of TiO_2_.

### 3.3. Microstructure, Crystalline Phase, and Chemical Composition Analysis of the TiO_2_ NTPC

**Figure 3:**
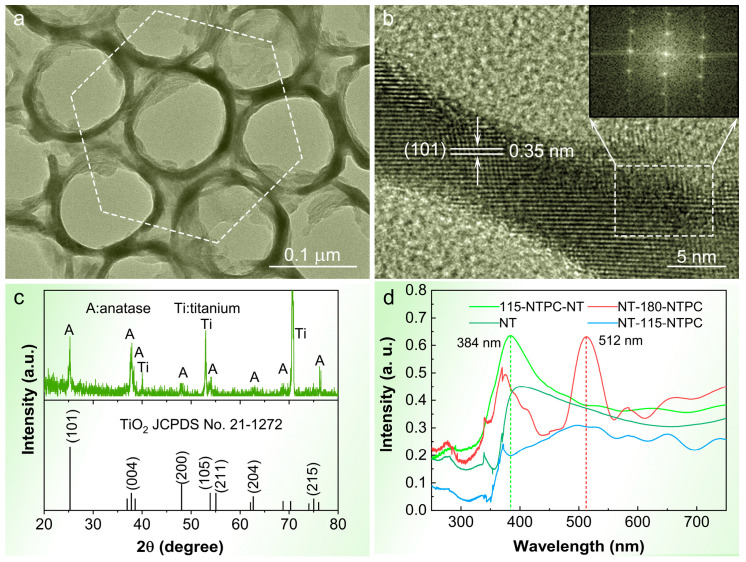
(**a**) A low-magnification FE-TEM image of the TiO_2_ 115 NTPC. (**b**) A HR-TEM image of the area highlighted by the white dashed hexagon in (**a**). Inset: Fast-Fourier Transform diffraction patterns of the areas bounded by the white dashed box in (**b**). (**c**) Corresponding XRD pattern. (**d**) Reflectance spectra of the TiO_2_ NT-115-NTPC, TiO_2_ NT-180-NTPC, TiO_2_ 115-NTPC-NT, and TiO_2_ NT infiltrated with electrolytes, respectively.

The microstructure, crystalline phase, and chemical composition of the TiO_2_ NTPCs are also analyzed by FE-TEM, XRD, and XPS. The low-magnification FE-TEM images further confirm that the TiO_2_ NTPC samples show a hexagonally arranged and bamboo-shaped periodic structure in the axial direction, which is consistent with FE-SEM results (Figure 3a,b and Figure S2). The HR-TEM image reveals that the well-resolved lattice spacing of 0.35 nm matches the d-spacing of the (101) plane of the anatase TiO_2_, which is further proved by the corresponding Fast-Fourier Transform diffraction pattern (inset of Figure 3b) [42,43]. Figure 3c presents the XRD pattern of the TiO_2_ NTPC, suggesting that all the diffraction peaks can be indexed to the anatase TiO_2_ (JCPDS 21-1276) except those from the Ti substrate [44,45,46]. The XPS spectra also demonstrate that the TiO_2_ NTPC samples are pure anatase with some oxygen deficiencies (Figure S3) [47].

### 3.4. Morphological Characterizaiton and Optical Obsorption Properties of the TiO_2_ NTPC Bi-Layer Structure

To confirm that the PBG reflection effect can lead to a significant enhancement in light absorption, we further fabricated the TiO_2_ NTPC bi-layer structure by successive two-step anodization. Figure S4 depicts the cross-sectional FE-SEM image of the TiO_2_ 115-NTPC bi-layer structure (referred as TiO_2_ NT-115-NTPC). The TiO_2_ NT-115-NTPCs with 15 periods can be clearly seen to seamlessly grow beneath the smooth-walled NT layer with a thickness of approximately 500 nm, ensuring excellent connection between the two layers and easy electrolyte infiltration. For comparison, we also fabricated a TiO_2_ 180-NTPC bi-layer structure (referred as TiO_2_ NT-180-NTPC), another TiO_2_ 115-NTPC bi-layer structure consisting of top NTPC layer and bottom NTs (TiO_2_ 115-NTPC-NT), and a TiO_2_ NT without a photonic crystal layer. To gain more realistic insights into the optical properties of TiO_2_ NT-115-NTPC, TiO_2_ NT-180-NTPC, TiO_2_ 115-NTPC-NT, and TiO_2_ NT, the reflectance spectra of the four samples in electrolytes were examined. As shown in Figure 3d, the TiO_2_ NT-115-NTPC exhibits the strongest UV light harvesting capacity among the aforementioned four samples. This could be mainly attributed to the Bragg mirror effect of the bottom TiO_2_ 115-NTPC with the PBG (3.2 eV) coinciding with the electronic bandgap of anatase TiO_2_ (3.2 eV) that can reflect the UV light back to the absorbing NT layer, thus leading to the boosted UV light absorption of the top TiO_2_ NT layer. Enhanced light absorption has been found in the TiO_2_ NTPC bi-layer structure-based dye-sensitized solar cells [48].

### 3.5. PEC Water Splitting Activity of the TiO_2_ NTPC Structure

**Figure 4:**
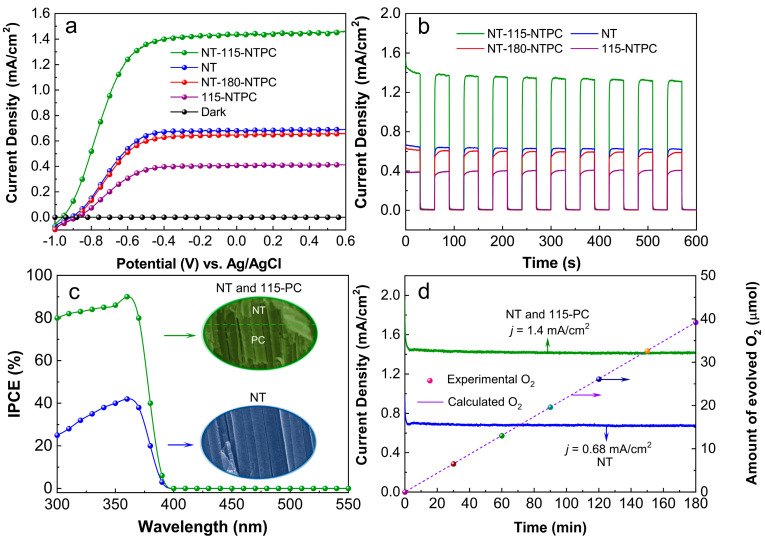
(**a**) Current vs. voltage (J−V) curves acquired from TiO_2_ NT-115-NTPC, TiO_2_ NT-180-NTPC, TiO_2_ 115-NTPC, TiO_2_ NT, respectively. (**b**) The corresponding transient photocurrent responses performed at 0.22 vs. Ag/AgCl. (**c**) The IPCE spectra of TiO_2_ NT-115-NTPC and TiO_2_ NT measured at an incident wavelength range from 300 nm to 550 nm at a potential 0.22 V vs. Ag/AgCl. (**d**) Photocurrent versus time (J−t) curves of TiO_2_ NT-115-NTPC and TiO_2_ NT obtained at 0.22 V vs. RHE. The dashed line and colorful spheres show the amount of evolved O_2_ calculated theoretically and detected experimentally for TiO_2_ NT-115-NTPC, respectively.

To determine the promoted PEC performance of TiO_2_ NT-115-NTPC, a set of PEC measurements were carried out in a three-electrode configuration using the as-prepared samples, Pt mesh, and Ag/AgCl (3 mol L^−1^ KCl-filled) as the working, counter, and reference electrodes, respectively. The electrolytes for the PEC water splitting reaction were an aqueous solution of 1M NaOH (pH = 13.6). Figure 4a displays the linear-sweep voltammogram (LSV) sweeps for TiO_2_ NT-115-NTPC, TiO_2_ NT-180-NTPC, TiO_2_ 115-NTPC, and TiO_2_ NT under light irradiation and dark conditions. All the samples produced almost negligible dark current in comparison with their photocurrent, suggesting no occurrence of electrocatalytic water splitting. Under irradiation, the photocurrent density of TiO_2_ NT-115-NTPC sharply increased and largely surpassed those of TiO_2_ NT-180-NTPC, TiO_2_ 115-NTPC, and TiO_2_ NT, which signifies that the TiO_2_ NT-115-NTPC had the highest PEC performance among the four samples. To elucidate this phenomenon more distinctly, their transient photocurrent responses were also measured under illumination with several 10 s light on/off cycles at 0.22 V vs. Ag/AgCl [1.23 V vs. RHE (reversible hydrogen electrode)], and the results are presented in Figure 4b.

At 0.22 V vs. Ag/AgCl, the TiO_2_ NT-115-NTPC delivered a maximal photocurrent density of 1.4 mA/cm^2^, and it was about 2.05, 2.15 and 3.5 times those of TiO_2_ NT TiO_2_ NT-180-NTPC and TiO_2_ 115-NTPC, respectively. The low photocurrent of the TiO_2_ NT-180-NTPC can be ascribed to the fact that its PBG position of the bottom TiO_2_ 180-NTPC was outside of the electronic bandgap of anatase TiO_2_ (3.2 eV), meaning that it could not reflect the UV light back to the top NT layer. That is to say, the bottom TiO_2_ 180-NTPC had no impact on the PEC performance of TiO_2_ NT-180-NTPC. Compared with TiO_2_ NT, the low photocurrent of the TiO_2_ 115-NTPC was mainly due to the single NTPC layer with a PGB position of 384 nm, which resulted in the decrease in UV light absorption.

To visualize the photocurrent enhancement owing to the promoted UV light absorption, incident photon-to-current conversion efficiency (IPCE) measurements were conducted on TiO_2_ NT-115-NTPC and TiO_2_ NT at 0.22 V vs. Ag/AgCl. The IPCE could be calculated as a percentage according to the following equation [49]:

(1) $$IPCE=\frac{1240I}{\lambda J_{light}}$$

where I is the measured photocurrent density at a specific wavelength, λ is the wavelength of the incident light, and $J_{light}$ is the light intensity of a specific wavelength. As shown in Figure 4c, the TiO_2_ NT-115-NTPC has greatly boosted IPCE values only in the UV region and reaches its highest value of 96% at 380 nm compared with TiO_2_ NT. The result provides a clue suggesting that the bottom TiO_2_ 115-NTPC makes a great contribution to PEC performance in the UV light region.

PEC stability and Faradic efficiency are two important parameters for the practical application of photoelectrode. Figure 4d presents the photocurrent–time curves of TiO_2_ NT-115-NTPC and TiO_2_ NT measured at 0.22 V vs. Ag/AgCl and continuous light illumination. The photocurrent densities of both samples are very stable, and there is no indication of deterioration during the entirely measured 3 h. To clarify whether the observed photocurrent originates from the oxygen evolution reaction, the fluorescence sensor is employed to determine the amount of oxygen evolved from the TiO_2_ NT-115-NTPC. The amount of evolved oxygen increases linearly with the illumination time with unity Faradic efficiency. In addition, the surface morphology and crystal phase of the TiO_2_ NT-115-NTPC remain intact after PEC water splitting for 3 h (Figure S5), illustrating that the TiO2 NT-115-NTPC possess prominent stability in the oxygen evolution reaction.

### 3.6. PEC Water Splitting Activity Mechanism

**Figure 5:**
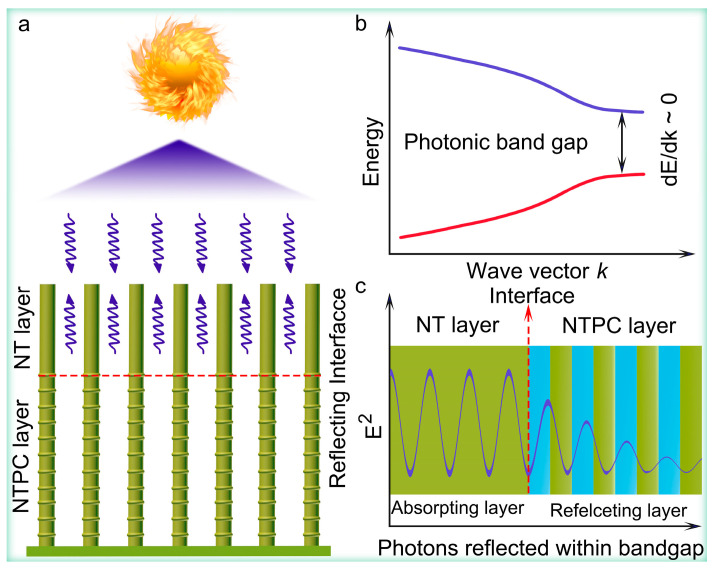
(**a**) A schematic drawing of mechanism of boosted UV light absorption of TiO_2_ NT-115 NTPC. Down arrow and up arrow represent the incident light and reflected light, respectively. (**b**) Schematic optical band structure of TiO_2_ nanotube photonic crystal. (**c**) Photons reflected within bandgap for further absorption.

Based on the above experimental results, the promoted PEC performance of TiO_2_ NT-115-NTPC can be mainly attributed to the significant enhancement of UV light absorption induced by the its bi-layer structure consisting of a top NT layer and a bottom NTPC layer (Figure 5a). As shown in Figure S6, the PBG (3.2 eV) of the bottom TiO_2_ 115-NTPC overlaps with the electronic bandgap of anatase TiO_2_ (3.2 eV). When the UV light strikes the TiO_2_ NT-115-NTPC, a portion of UV light is absorbed by the top TiO_2_ NT, producing the photoexcited electrons and holes, whereas another portion of UV light penetrates the top TiO_2_ NT layer and is reflected by the bottom the 115-NTPC layer serving as the Bragg mirror. In such cases, the reflected light can be absorbed again by the top TiO_2_ NT, hence promoting UV light absorption by the top TiO_2_ NT. Additionally, optical interference occurs when UV light is being transmitted and reflected, which leads to strong UV photon resonance modes in the top NT absorbing layer, thus also boosting UV light absorption by the top TiO_2_ NT (Figure 5b,c). Similar phenomena have been confirmed for other opal photonic crystal photocatalysis [50,51,52]. Accordingly, more photoexcited electrons and holes are generated, and remarkable promotion of PEC performance is achieved for the TiO_2_ NT-115-NTPC.

## 4. Conclusions

In summary, we designed and fabricated a novel TiO_2_ NTPC bi-layer structure photoanode consisting of a top NT layer and a bottom NTPC layer. In this architecture, when the PBG of bottom NTPC overlapped the with electronic bandgap of TiO_2_, the bottom TiO_2_ NTPC produced the Bragg mirror effect, leading to boosted UV light harvesting of top TiO_2_ NT layer. Benefiting from promoted UV light absorption, the TiO_2_ NT-115-NTPC yielded a photocurrent density of 1.4 mA/cm^2^ at 0.22 V vs. Ag/AgCl with a Faradic efficiency of 100%, nearly two times higher than that of conventional TiO_2_ NT. Furthermore, IPCE was also promoted within UV light region. This work provides an effective strategy for improving PEC water splitting through intensifying light–matter interaction.

## Data Availability

Data are available from the authors on request.

## Supplementary Materials

The following supporting information can be downloaded at https://www.mdpi.com/article/10.3390/nano14211695/s1, Figure S1: Photographs of the TiO_2_ 115-NTPC in air and infiltrated with ethanol, respectively. Figure S2: (a) A low-magnification FE-TEM image of the TiO_2_ 180-NTPC. (b) An HR-TEM image of the area highlighted by the white dashed box in (a). (c) Fast-Fourier Transform diffraction patterns of the areas bounded by the white dashed box in (b). (d) Projected atomic models in [101] directions. Figure S3: (a) The XPS survey spectra of the TiO_2_ NT-115-NTPC. (b) The corresponding normalized Ti 2p XPS spectra and normal O 1s XPS spectra. Figure S4: FE-SEM images of the TiO_2_ NT-115-NTPC. Figure S5: (a) A FE-SEM image of the TiO_2_ NT-115-NTPC after undergoing the PEC water splitting reaction for 180 min. (b,c) The corresponding FE-TEM image.
