# Supplementary material for: Exploiting the Bragg Mirror Effect of TiO2 Nanotube Photonic Crystals for Promoting Photoelectrochemical Water Splitting
Source: Nanomaterials (Basel). 2024 Oct 23;14(21):1695. doi: 10.3390/nano14211695 (PMC11547193; doi:10.3390/nano14211695)
Supplement: Supplementary file 1 [file nanomaterials-14-01695-s001.zip › nanomaterials-3220749-supplementary.pdf]

Supporting Information

Exploiting the Bragg Mirror Effect of TiO_2 Nanotube Photonic Crystals for Promoting Photoelectrochemical Water Splitting

Ming Meng[1,*], Hucheng Zhou[1], Jing Yang[1], Liwei Wang[1], Chunyang Li[1], Honglei Yuan[1], Yanling Hao[2,*] and Zhixing Gan[3,*]

[1] School of Physics and Telecommunication Engineering, Zhoukou Normal University, Zhoukou 466001, P. R. China; 20182013@zknu.edu.cn (H.Z); yangjing0410@zju.edu.cn (J.Y); wangliwei@zknu.edu.cn (L.W); yhl@zknu.edu.cn (H.Y)
[2] Key Laboratory for Micro-nano Functional materials of Qianxinan, Minzu Normal University of Xingyi, Xingyi, Guizhou, 56240, P. R. China
[3] Center for Future Optoelectronic Functional Materials, School of Computer and Electronic Information/School of Artificial Intelligence, Nanjing Normal University, Nanjing 210023, P. R. China
* Correspondence: mengmign@zknu.edu.cn (M.M); haoyanling@xynun.edu.cn (Y.H); zxgan@njnu.edu.cn (Z. G)

Citation: Meng, M.; Zhou, H.; Yang, J.; Wang, L.; Yuan, H.; Hao, Y. Gan, Z. Exploiting the Bragg mirror effect of TiO_2 nanotube photonic crystals for promoting photoelectrochemical water splitting. *Nanomaterials* **2024**, *X*, x. https://doi.org/10.3390/xxxxx

Academic Editor: First name Lastname

Received: date
Accepted: date
Published: date

Publisher's Note: MDPI stays neutral with regard to jurisdictional claims in published maps and institutional affiliations.

Copyright: © 2024 by the authors. Submitted for possible open access publication under the terms and conditions of the Creative Commons Attribution (CC BY) license (https://creativecommons.org/licenses/by/4.0/).

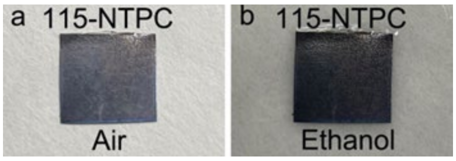

Figure S1. Photographs of the TiO_2 115-NTPC in air and infiltrated with ethanol, respectively.

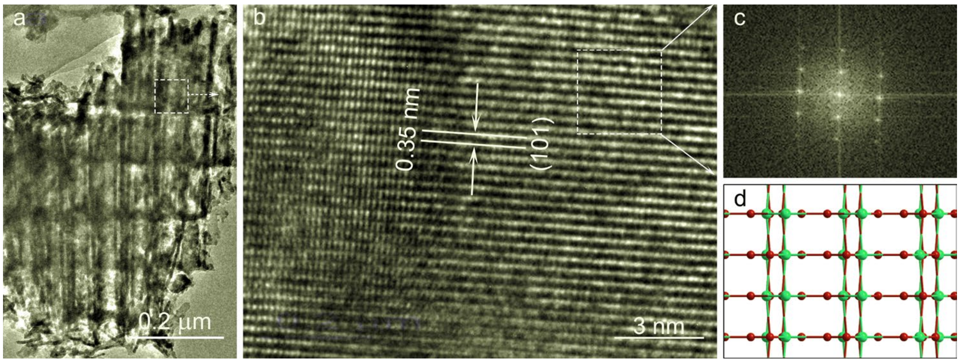

Figure S2. (a) Low-magnification FE-TEM image of the TiO_2 180-NTPC. (b) HR-TEM image of the area highlighted by the white dashed box in (a). (c) Fast-Fourier-Transform diffraction patterns of the areas bounded by the white dashed box in (b). (d) Projected atomic models along [101] directions.

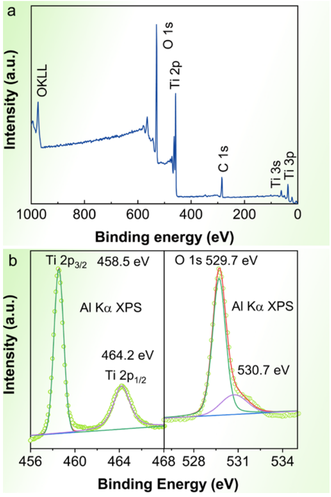

Figure S3. (a) XPS survey spectra of the TiO_2 NT-115-NTPC. (b) Corresponding the normalized Ti 2p XPS spectra and normal O 1s XPS spectra.

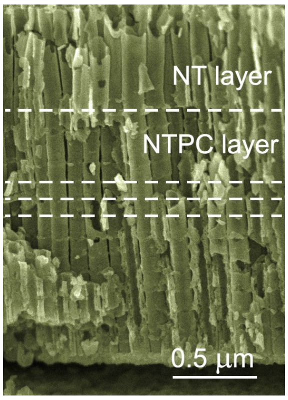

Figure S4. FE-SEM images of the TiO_2 NT-115-NTPC.

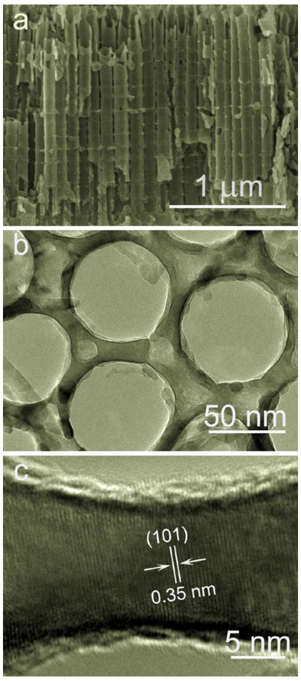

Figure S5. (a) FE-SEM image of the TiO_2 NT-115-NTPC after undergoing the PEC water splitting reaction for 180 min. (b-c) Corresponding FE-TEM image.

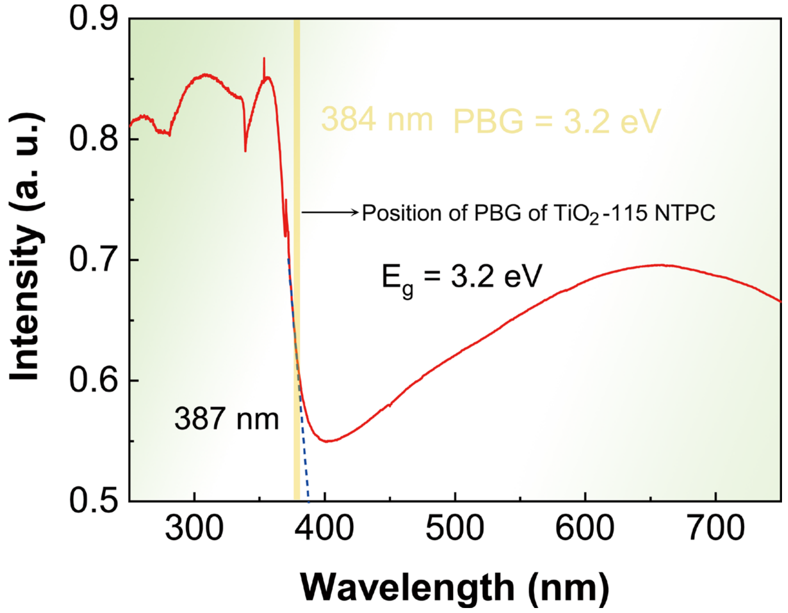

Figure S6 UV-visible Absorption spectrum of conventional TiO_2 nanotube arrays.
